# Pharmacodynamic Response to Anti-thyroid Drugs in Graves' Hyperthyroidism

**DOI:** 10.3389/fendo.2020.00286

**Published:** 2020-05-12

**Authors:** Ali Abbara, Sophie A. Clarke, Rosalind Brewster, Alexia Simonnard, Pei Chia Eng, Maria Phylactou, Deborah Papadopoulou, Chioma Izzi-Engbeaya, Amir H. Sam, Florian Wernig, Eliza Jonauskyte, Alexander N. Comninos, Karim Meeran, Tom W. Kelsey, Waljit S. Dhillo

**Affiliations:** ^1^Section of Endocrinology and Investigative Medicine, Division of Diabetes, Endocrinology and Metabolism, Hammersmith Hospital, Imperial College London, London, United Kingdom; ^2^Department of Endocrinology, Imperial College Healthcare NHS Trust, London, United Kingdom; ^3^School of Computer Science, University of St Andrews, St Andrews, United Kingdom

**Keywords:** Graves' disease, anti-thyroid drug, carbimazole, hyperthyroidism, dose

## Abstract

**Objective:** Graves' disease is the commonest cause of hyperthyroidism in populations with sufficient dietary iodine intake. Anti-thyroid drugs (ATD) are often used as the initial treatment for Graves' hyperthyroidism, however there is a paucity of data relating the dose of ATD therapy to the effect on thyroid hormone levels, increasing the risk of both over- and under-treatment. We aimed to determine the pharmacodynamic response to the ATD carbimazole.

**Design:** Retrospective cohort study.

**Methods:** Participants were patients (*n* = 441) diagnosed with Graves' disease at Imperial College Healthcare NHS Trust between 2009 and 2018. The main outcome measure was change in thyroid hormone levels in response to ATD.

**Results:** Baseline thyroid hormone levels were positively associated with TSH receptor antibody titres (*P* < 0.0001). Baseline free triiodothyronine (fT3) were linearly related to free thyroxine (fT4) levels in the hyperthyroid state (fT3 = fT4^*^0.97–11), and fell proportionately with carbimazole. The percentage falls in fT4 and fT3 per day were associated with carbimazole dose (*P* < 0.0001). The magnitude of fall in thyroid hormones after the same dose of carbimazole was lower during follow up than at the initiation visit. The fall in thyroid hormone levels approximated to a linear response if assessed at least 3 weeks after commencement of carbimazole. Following withdrawal of antithyroid drug treatment, the risk of relapse was greater in patients with higher initial fT4, initial TSH receptor antibody titre, males, smokers, and British Caucasian ethnicity.

**Conclusion:** We identify a dose-response relationship for fall in thyroid hormones in response to carbimazole to aid in the selection of dose for Graves' hyperthyroidism.

## Introduction

Overt hyperthyroidism affects 1.3% of people in iodine-replete populations (1) and if untreated is associated with a catabolic state characterized by weight loss, reduced bone mineral density, atrial fibrillation, and thromboembolic events (2, 3). Graves' disease is the commonest cause of hyperthyroidism accounting for up to 80% of cases (4–6), with a lifetime prevalence of 3% in women and 0.5% in men (1, 7).

Recent evidence suggests that rapid control of hyperthyroidism in Graves' disease, whilst avoiding over-treatment, is desirable. A low thyroid stimulating hormone (TSH) level at 1 year following diagnosis of Graves' disease was associated with a 55% increase in cardiovascular mortality independent of treatment modality (8). Similarly, every 6 months' duration with suppressed TSH levels in patients with hyperthyroidism was associated with a 11–13% increase in total mortality (9). Conversely, even transient hypothyroidism during treatment has been associated with greater weight-gain than those without over-treatment following anti-thyroid medications (10). Moreover, avoidance of hypothyroidism is recommended to prevent exacerbation of thyroid eye disease (11). Thus, the prompt and sustained normalization of thyroid hormone levels is of foremost importance in the management of patients diagnosed with Graves' disease (7).

Treatment modalities for the management of Graves' hyperthyroidism include anti-thyroid drugs (ATD), radioactive iodine (RAI) therapy, or total thyroidectomy. ATD is favored as first line therapy in Europe, with remission achieved in approximately half of patients after a 12–18 month duration of treatment (6, 12, 13). Traditionally, radioactive iodine has been preferred in USA (7), although recent American Thyroid Association (ATA) guidelines have suggested that ATD can also be considered as first line (14). However, a pharmacodynamic relationship between ATD and thyroid hormone levels has yet to be clearly described.

Thionamides inhibit the thyroid peroxidase enzyme to reduce thyroid hormone synthesis (15). In the UK, the two predominant ATDs used are carbimazole (which is entirely metabolized to methimazole), and propylthiouracil (PTU). Methimazole has a longer half-life (t_1/2_ 4–6 h) than PTU (t_1/2_ 75 min), enabling once-daily administration, whereas PTU is given as multiple doses over a day (15, 16). Blood levels of both drugs peak 1–2 h following ingestion, with inhibition of thyroid hormone synthesis lasting for 12–24 h following PTU (17) and >24 h following methimazole (15, 18). ATDs may be given either via a “dose titration” regimen whereby initial higher doses are reduced over time, or as a “block and replace” regimen using fully inhibitory doses of ATDs with concomitant thyroxine replacement to maintain euthyroidism. Neither approach has been reliably demonstrated as superior in achieving remission (19). However, the dose-titration regimen is associated with lower doses of ATDs, and thus a potentially reduced risk of dose-related side-effects such as agranulocytosis (20). Likewise, rates of discontinuation due to side-effects from ATDs are lower following the “dose-titration” method compared to “block and replace” (19–22).

To date, there is a paucity of evidence to describe the pharmacodynamic response between the dose of ATD and the resultant reduction of thyroid hormone levels. Consequently, many clinicians adopt experience-based strategies to prescribe ATDs. For example, Abraham and colleagues recommend initiating carbimazole/methimazole with a dose of 10–20 mg once daily if fT4 is <40 pmol/l and 40 mg once daily if fT4 is >40 pmol/l and then halving the dose following 1 month of treatment (23).

In summary, Graves' disease is one of the most common endocrine pathologies encountered by the endocrinologist, and whilst medical therapy with ATD is often adopted as the first line treatment modality, there is scarce data to support physicians in selecting the dose of carbimazole for initiation and subsequent dose-titration. In the present study, we aimed to determine the pharmacodynamic relationship between dose of carbimazole and resultant change in thyroid hormone levels. We also investigated baseline factors that could predict the chance of spontaneous remission following ATDs in a UK population to inform the likely success of medical therapy.

## Materials and Methods

### Study Design

We undertook a retrospective analysis of patients attending Imperial College Healthcare NHS Trust between 2009 and 2018, who had been diagnosed with Graves' disease and treated with anti-thyroid drugs. This audit was registered with Imperial College NHS Healthcare Trust (registration number ASM_024).

### Study Participants

Participants had a diagnosis of hyperthyroidism due to Graves' disease confirmed by: biochemical hyperthyroidism and positive thyroid stimulating hormone (TSH) receptor autoantibody (>0.3 U/mL) or a technetium thyroid scan consistent with Graves' disease. Biochemical hyperthyroidism was defined as a supressed TSH (<0.3 mU/L), elevated free thyroxine (fT4) (>23.0 pmol/L), and/or elevated free triiodothyronine (fT3) (>5.7 pmol/L). Exclusion criteria included patients with thyrotoxicosis but unconfirmed Graves' disease, and those with dual thyroid pathology, and patients who were pregnant at the time of diagnosis. Demographic data, initial thyroid hormone levels, thyroid antibody titres, doses of ATDs, follow-up thyroid hormone levels, and clinical course were reviewed.

### Standard Care at Our Centre

Patients included in this study were treated with a dose-titration regimen. The highest initial dose for commencing treatment was 40 mg daily for patients with elevated thyroid hormone levels. A lower dose without clear parameters was used for patients with lower levels of thyroid hormones based on individual endocrinology physician's practice. Patients are usually reviewed every 4–6 weeks during the first few months of treatment until thyroid hormone levels have stabilized. Treatment is continued for at least 18 months before withdrawal of ATD medication is trialed. Patients have measurement of their thyroid hormone levels 4 weeks after cessation of ATDs. Those that have normal thyroid function tests at this juncture are discharged with a recommendation for infrequent monitoring in primary care (e.g. 6 monthly, or sooner if symptoms recur). If thyroid function testing suggests persistence/relapse of thyrotoxicosis, antithyroid drug therapy is restarted, and the patient is recommended to have definitive management with radioiodine therapy or surgery. Both those with persistence of disease on withdrawal and those with initial remission but subsequent relapse were included in the assessment of patients with relapse, with at least 3 months' follow-up following drug cessation. The weightings for individual aspects of the relapse rate score were assigned according to the frequency of relapse in the indicated subgroups.

### Hormonal Assay Methodology

Serum TSH, fT4 and fT3 levels were measured using automated chemiluminescent immunoassays (Abbot Diagnostics, Maidenhead, UK). Interassay coefficients of variation were: TSH ≤5.3%, fT4 ≤6.3%, and fT3 ≤5%. Limits of detection for each assay were as follows: TSH ≤0.0025 mU/l, fT4 5.15 pmol/l, fT3 1.536 pmol/l. TSH receptor antibody titre was measured using third generation ELISA kit (RSR laboratories, Cardiff, United Kingdom) with interassay coefficient of variation of 8.8% and lower limit of detectability of 0.08 u/L. TPO antibody titre was measured using ELISA (Orgentecs) with interassay coefficient of variation of 8.5% and a measuring range of 0–3,000 U/ml.

### Statistical Analysis

Data was analyzed using GraphPad PRISM version 7.0 and STATA version 14.0. Data distribution was assessed using D'Agostino and Pearson normality test. Parametric data was presented as mean ± standard deviation (SD), whereas non-parametric data was presented as median with interquartile range (IQR). Non-parametrically distributed data were compared using Kruskal-Wallis test with *post-hoc* Dunn's, or Mann-Whitney *U*-tests. Data with multiple groups parametrically distributed was compared by one-way analysis of variance (ANOVA). Categorical data were compared using logistic regression. Simple linear regression was used to determine the association between two variables. Multivariable linear regression was used to assess the association of TSH receptor antibody titre, TPO antibody titre, smoking status, ethnicity, and gender with serum fT4 levels. Multivariable logistic regression was used to assess the effect on remission of the following baseline variables: gender, smoking, ethnicity, TPO antibody status, TSH receptor antibody titre, initial fT4 level, and final fT4 level. A *P* < 0.05 was regarded as statistically significant.

The daily dose-response relationship for fall in thyroid hormones in response to carbimazole was derived from linear regression models for the percentage fall in fT4 and fT3 levels between visits. The formula used is fT4(*n*+1) = fT4(*n*) – (m^*^Dose^*^fT4(day *n*)+c), where fT4(*n*) is the level on day *n* after initial visit; Dose is the carbimazole taken each day between visits; m is the slope of the linear model determined from the data; c is the intercept of the same model. The formula for fT3 is the same. Slope and intercept values were adjusted for weight at initial visit. Since, for constant dose, the fall in fT4 and fT3 levels between first and second visit is bigger than the fall between second and third visits and between third and fourth visits, we refined the initial model to incorporate (a) slope and intercept values derived from regression analysis for the time between each visit and (b) a dose exponent factor that accounts for the increased fall. Hence the overall model for is fT4(*n*+1) = fT4(*n*) – (m^*^Dose^p*^fT4(day *n*) + c), with p representing the dose exponent and all other variable definitions unchanged. Absolute measure of model fit is reported as root mean square error (RMSE) in pmol/L, the square root of the variance of the residuals, with zero indicating a perfect fit.

## Results

### Baseline Characteristics

We reviewed medical records of 526 patients with thyrotoxicosis and identified 441 patients with a confirmed diagnosis of Graves' disease. Mean (±SD) age was 44 (±15) years and 81% of patients were female (Table 1). Patients were followed up for a median duration of 328 days (range 7–1,443 days). TSH receptor antibody titres were recorded for 99.1% (*n* = 437) of patients with a median titre of 4.1 U/ml (IQR 1.95–8.0 U/ml). TPO antibodies were measured in 59.0% (*n* = 260) of patients (median 139, IQR 0–385 U/ml).

**Table 1:** Baseline characteristics of patients with Graves' disease.

	***N***
Total number of patients reviewed	526
Total number of patients with complete records	441
Follow up duration (days)	328 (158–544)
Age (years)	44 (± 15)
**Sex**
Male (%)	82 (18.6%)
Female (%)	358 (81.4%)
**Ethnicity**
Black (African and Caribbean) (%)	50 (11.4%)
White (Caucasian) (%)	159 (36.1%)
Asian (%)	47 (11.0%)
Mixed (%)	10 (2.3%)
Other (%)	87 (20.0%)
Unknown (%)	87 (20.0%)

*Continuous variables displaying parametric distribution are presented as mean ± standard deviation (SD), whereas non-parametric variables are presented as median with interquartile range (IQR)*.

### Baseline Thyroid Hormone Levels

**Figure 1:**
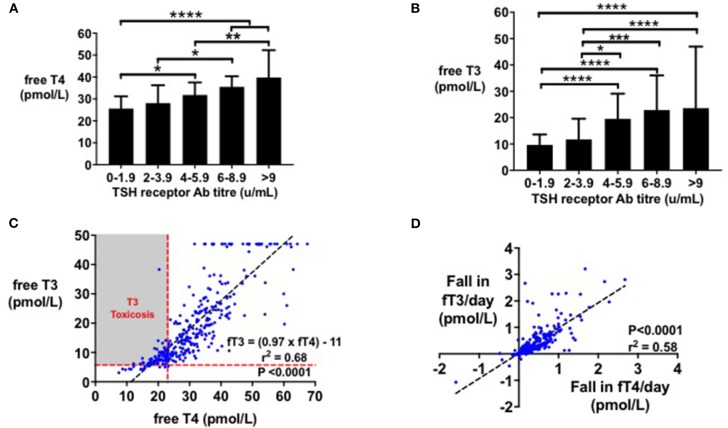
**(A)** Bar graph (median with IQR) of initial fT4 (pmol/l) by initial TSHR antibody titre (mU/l). Initial fT4 (pmol/l) increased with TSHR antibody titre (*P < 0.05, **P < 0.01, ****P < 0.0001). Initial fT4 levels were predicted by initial TSHR antibody titre (*r*^2^ = 0.11, *P* < 0.0001). **(B)** Bar graph (median with IQR) of initial fT3 (pmol/l) by initial TSHR antibody titre (mU/l). Initial fT3 (pmol/l) increased with TSHR antibody titre (*P < 0.05, ***P < 0.001, ****P < 0.0001). Initial fT3 levels were predicted by initial TSHR antibody titre (*r*^2^ = 0.12, *P* < 0.0001). **(C)** Scattergraph of initial fT4 (pmol/l) by initial fT3 (pmol/l). Initial fT4 and fT3 increased in an ~1:1 ratio, with fT4 predicting fT3 levels (fT3 = (0.97 × fT4)-11, *r*^2^ = 0.68, *P* < 0.0001). **(D)** Scattergraph of fall in fT3 (pmol/l) per day by fall in fT4 (pmol/l) per day, following 4 weeks' of treatment with carbimazole. Fall in fT4 per day (pmol/l) predicted fall in fT3 per day (pmol/l) by the equation: Fall in fT3 per day = 0.9541* Fall in fT4/day + 0.02, *r*^2^ = 0.58, *P* < 0.0001.

FT4 levels and fT3 levels were associated with TSH receptor antibody titre (*r*^2^ = 0.11 and *r*^2^ = 0.12, respectively, *P* < 0.0001). Initial fT4 (Figure 1A) and fT3 levels (Figure 1B) were higher in patients with greater TSH receptor antibody titres at diagnosis (*P* < 0.0001). The ratio of fT4:fT3 before treatment was ~2:1, and fT3 could be predicted from fT4 levels using the equation fT3 = 0.97^*^fT4-11, (*r*^2^ = 0.68, *P* < 0.0001) (Figure 1C). Age, sex, ethnicity, and smoking status were not associated with initial fT4 levels by linear regression.

### Change in Thyroid Hormone Levels in Response to Carbimazole

**Figure 2:**
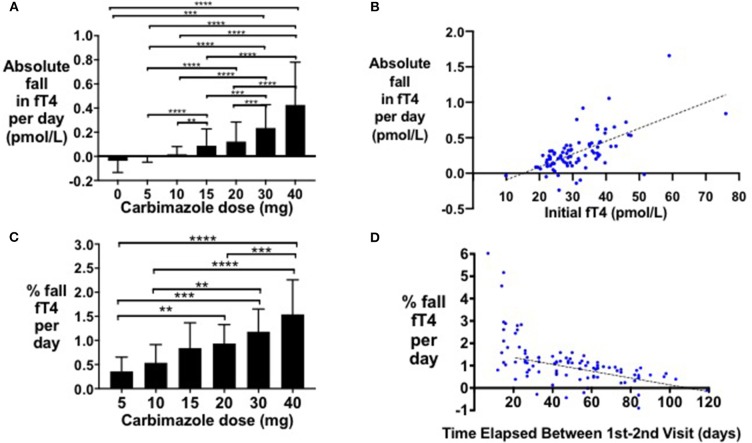
**(A)** Bar graph of absolute fall in fT4 per day by carbimazole dose across all study visits (**P < 0.01, ***P < 0.001, ****P < 0.0001). **(B)** Scattergraph of absolute fall in fT4 per day (pmol/L) in patients receiving 20 mg carbimazole daily following initial visit (absolute fall in fT4 per day = 0.018*initial fT4 −0.27, *r*^2^ = 0.40, *P* < 0.0001). **(C)** Scattergraph of percentage fall in fT4 per day (%) between the first and second visits in patients receiving 20 mg carbimazole daily following their initial assessment (% fall in fT4 per day = −0.015 * number of days between visits + 1.693, *r*^2^ = 0.30, *P* < 0.0001). **(D)** Scattergraph of percentage fall in fT4 per day (%) in patients taking 20 mg carbimazole by time elapsed (days) between the first and second visits.

Following initiation of antithyroid treatment with carbimazole, both fT3 and fT4 decreased proportionately in a 1:1 ratio (*r*^2^ = 0.58, *P* < 0.0001) (Figure 1D). As a first pass analysis, we plotted the absolute fall in fT4 per day by dose of carbimazole for all visits (Figure 2A). We then selected only patients receiving 20 mg of carbimazole to demonstrate that the absolute fall in fT4 per day was proportional to the baseline fT4 level prior to commencing that dose (Figure 2B). Thus, we used fall in fT4 from baseline expressed as a percentage for subsequent analyses (Figure 2C). We examined the interval required for reliable assessment of percentage change in fT4 following each dose of carbimazole. We observed that the relationship between the fall in fT4 and time is curvilinear with a rapid fall during the first 21 days following initiation of 20 mg of carbimazole (Figure 2D). However, the percentage fall in fT4 per day approximates to a linear relationship after 21 days and thus for subsequent analyses of dose response with carbimazole we excluded data with intervals shorter than 21 days. The relationship between fall in fT4 and dose of carbimazole was altered by duration from first commencement of carbimazole. Indeed, the percentage fall in fT4 per day after the same dose of carbimazole (20 mg) was reduced in patients with a longer duration since initial presentation (*r* = −0.015, *r*^2^ = 0.29, *P* < 0.0001).

### Pharmacodynamic Response to Dose of Carbimazole

**Figure 3:**
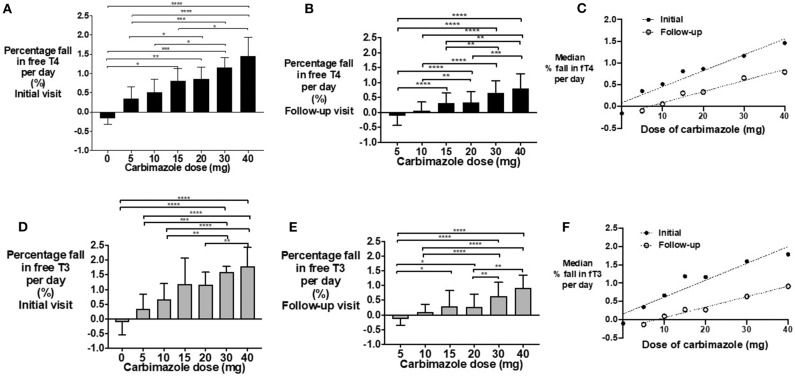
**(A)** Bar graph of percentage fall in fT4 per day in patients on carbimazole following their initial visit with groups compared using Kruskal-Wallis test with *post-hoc* Dunn's test (*P < 0.05, **P < 0.01, ***P < 0.001, ****P < 0.0001). **(B)** Bar graph of percentage fall in fT4 per day in patients on carbimazole following their follow-up visit with groups compared using Kruskal-Wallis test with *post-hoc* Dunn's test (*P < 0.05, **P < 0.01, ***P < 0.001, ****P < 0.0001). **(C)** Scattergraph of median percentage fall in fT4 per day in patients receiving carbimazole at both initial and follow-up visits. Initial median % fall in fT4 per day = 0.04* carbimazole dose + 0.09, *r*^2^ = 0.93, *P* = 0.0004. Follow-up median% fall in fT4 per day = 0.03*carbimazole dose −0.17, *r*^2^ = 0.97, *P* = 0.0005. **(D)** Bar graph of percentage fall in fT3 per day in patients on carbimazole following their initial visit with groups compared using Kruskal-Wallis test with *post-hoc* Dunn's test (*P < 0.05, **P < 0.01, ***P < 0.001, ****P < 0.000). **(E)** Bar graph of percentage fall in fT3 per day in patients on carbimazole following their follow-up visit with groups compared using Kruskal-Wallis test with *post-hoc* Dunn's test (*P < 0.05, **P < 0.01, ***P < 0.001, ****P < 0.0001). **(F)** Scattergraph of median percentage fall in fT3 per day in patients receiving carbimazole at both initial and follow-up visits. Initial median% fall in fT3 per day = 0.05* carbimazole dose + 0.16, *r*^2^ = 0.91, *P* = 0.0008. Follow-up median% fall in fT3 per day = 0.03* carbimazole dose −0.23, *r*^2^ = 0.98, *P* = 0.0002.

The percentage fall in fT4 per day was associated with carbimazole dose both at initial (Figure 3A) and follow-up assessments (Figure 3B). Notably, the median percentage reduction in fT4 levels was lower at follow-up assessment compared to initial assessment (Figure 3C). A similar dose-response relationship was observed for fT3 levels (Figures 3D–F) and for absolute reductions in thyroid hormone levels (Supplemental Figure 1). Neither gender (*P* = 0.61), nor smoking status (*P* = 0.17), influenced thyroid hormonal response to carbimazole. Similarly, neither body-weight (*P* = 0.35), nor splitting the dose of carbimazole during the day (*P* = 0.36) influenced the fall in fT4 levels following carbimazole.

The daily dose-response relationship for fall in thyroid hormones in response to carbimazole formula is fT4(*n* + 1) = fT4(*n*) – (m^*^Dose^*^fT4(day *n*) + c), where fT4(*n*) is the level on day n after initial visit; Dose is the carbimazole dose taken each day between visits; m is the slope of the linear model determined from the data; c is the intercept of the same model. Although body-weight was not a significant factor in regression modeling over several weeks, adjustment of the slope, and intercept values depending on body-weight at first visit slightly increases the conformity of the dose-response model to observations (root mean square error RMSE when comparing observations to predictions falling from 4.44 to 4.28 pmol/L for fT4 and from 3.01 to 2.67 pmol/L for fT3). The derived slope and intercept values are given in Supplemental Table 1. The power model derived to account for greater fall in fT4 and fT3 levels for a given dose after the first and second visit has similar RMSE. The models have been implemented as a web application, available at https://wkapps.host.cs.st-andrews.ac.uk/Graves.

### Time to Achieve Euthyroid Status and Risk of Overtreatment

In patients with at least 2 months' duration of treatment (*n* = 422), the majority (95%) achieved normal fT4 and fT3 levels (95 and 74%, respectively). Euthyroid status, defined as having TSH, fT4, and fT3 all within range, was achieved by 28% of patients at a median time of 192 days (range 84–407 days). Similarly, 29% (*n* = 98) of patients were over-treated and rendered hypothyroid as indicated by either a TSH >4.2 mU/l, or fT4 <9 pmol/l. Greater initial carbimazole dose (*P* = 0.04) and higher initial fT4 level (*P* = 0.04) increased the risk of over-treatment when assessed by univariate logistic regression, whereas initial TPO antibody titre (*P* = 0.09), TSH receptor antibody titre (*P* = 0.79), sex (*P* = 0.23), ethnicity (*P* = 0.40), age (*P* = 0.86), and smoking status (*P* = 0.36) were not significant predictors.

### Risk Factors for Relapse

Of the study cohort, 120 patients had completed 18 months' of antithyroid treatment and had a trial off treatment with carbimazole. Of these, 19% (*n* = 23) had evidence of relapse/persistent disease at the first clinical assessment following cessation of antithyroid medication, and a further 16% (*n* = 19) had relapse at subsequent assessments with median time to relapse being 85 days (range 25–335 days). Thus, 35% (*n* = 42) in total had relapse following cessation of medical therapy.

**Figure 4:**
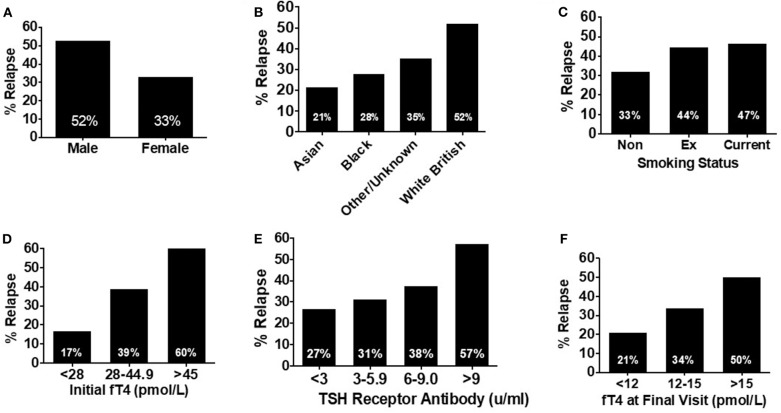
**(A)** Bar graph showing relapse rates in patients receiving at least 18 months' of treatment (*n* = 120) by sex. Male patients were nearly 1.5 times more likely to experience relapse compared with female patients. **(B)** Bar graph showing relapse rates in patients receiving at least 18 months' of treatment (*n* = 120) by ethnicity. Relapse was most likely in patients of white British origin, having an almost 4-fold increased risk of relapse compared with Asian patients. **(C)** Bar graph showing relapse rates in patients receiving at least 18 months' of treatment (*n* = 120) by smoking status. Risk of relapse was over 50% more likely in patients who had ever smoked, compared to those who had never smoked. **(D)** Bar graph showing relapse rates in patients receiving at least 18 months' of treatment (*n* = 120) by initial fT4. Patients with an initial fT4 >45 pmol/l were a third more likely to experience relapse, than those with an initial serum fT4 <23 pmol/l. **(E)** Bar graph showing relapse rates in patients receiving at least 18 months' of treatment (*n* = 120) by initial TSH receptor antibody titre. There was a trend to increasing risk of relapse as initial TSH receptor antibody titre increases with those with a titre >9 mU/l having a >2-fold increase in risk of relapse, compared to those with an initial TSH receptor antibody titre <2 mU/l. **(F)** Bar graph showing relapse rates in patients receiving at least 18 months' of treatment (*n* = 120) by serum fT4 (pmol/l) at the final visit. As serum fT4 increased, so did the risk of relapse, with patients with a serum fT4 in the upper range of normal (>15 pmol/l) having a relapse rate more than twice that of those with a final serum fT4 in the lower part of the reference range (<12 pmol/l).

An increased frequency of relapse was observed in males (Figure 4A), white British ethnic origin (Figure 4B), and current or previous smoking (Figure 4C) (Table 2). Those with an initial fT4 >45 pmol/l had an increased odds of relapse compared to those with a fT4 <28 pmol/l (OR 7.5, 95% CI 1.69–33.27) (Figure 4D). Patients with a greater TSH receptor antibody titre at diagnosis also had an increased odds of relapse (OR 3.69 if TSHrAb >9 vs. <3 mU/L, 95% CI 1.32–10.29) (Figure 4E) (Table 2). Similarly, patients with a higher fT4 at the final visit prior to withdrawal of carbimazole had an increased odds of relapse (Figure 4F; OR 3.41 if fT4 >15 pmol/l vs. <12 pmol/l, 95% CI 1.07–10.87) (Table 2). In an adjusted multivariable logistic regression model (*r*^2^ = 0.09, *P* = 0.04) including ethnicity, gender, age, smoking status, TSHR and TPO antibody titre, and initial fT4 measurement, only initial fT4 significantly predicted risk of relapse (*P* = 0.02).

**Table 2:** Risk factors for relapse.

**Risk Factor**	**% of patients with relapse**	**Odds ratio**	**95% CI**	***P*-value**	**Adjusted odds ratio**	**95% CI**	***P*-value**
**Sex**				0.11			0.11
Male	52%						
Female	33%	0.44	0.16–1.20		0.38	0.12–1.23	
**Ethnicity**				0.18			
Asian	21%						
Black	28%	1.41	0.27–7.27	0.68	7.05	0.91–54.64	0.06
Other	35%	2.00	0.49–8.10	0.33	1.40	0.23–8.67	0.72
White	52%	3.92	0.90–17.08	0.07	3.67	0.68–19.92	0.13
**Smoking status**				0.41			
Non-smoker	32%						
Ex smoker	44%	1.70	0.59–4.89	0.32	2.30	0.60–8.79	0.22
Current smoker	47%	1.86	0.60–5.76	0.28	0.54	0.10–2.88	0.47
**TSHR titre (u/mL)**				0.09			
<3	27%						
3–5.9	31%	1.24	0.48–3.26	0.66	0.96	0.25–3.61	0.95
6–9.0	38%	1.66	0.61–4.49	0.32	3.16	0.78–12.81	0.11
>9	57%	3.69	1.32–10.29	0.01	7.95	1.96–32.24	0.004
**Free T4 (pmol/L) at initial visit**				0.02			
<28	17%						
28–44.9	39%	3.2	0.92–11.1	0.07	1.38	0.47–4.04	0.55
>45	60%	7.5	1.69–33.27	0.0008	2.39	0.29–19.84	0.42
**Free T4 (pmol/L) at final visit**				0.12			
<12	21%						
12–15	34%	2.81	0.58–13.7	0.20	3.00	0.65–13.83	0.16
>15	50%	3.41	1.07–10.87	0.05	5.67	0.85–37.62	0.07

*Rates (%) of relapse are presented for risk factors investigated. Rates of relapse were compared using logistic regression, with both adjusted and unadjusted odds ratios presented*.

**Figure 5:**
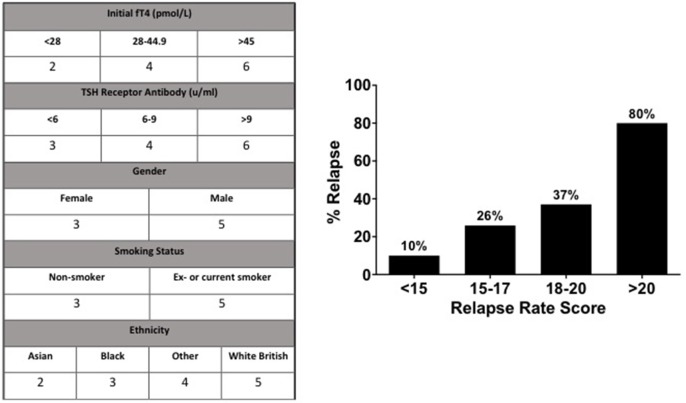
According to their relationship to risk of relapse, values were assigned to parameters to devise the “Relapse Rate Score”. Observed relapse increased with relapse rate score, such that those with a score >20 had an 80% chance of relapse, compared to 10% chance of relapse in patients with a relapse rate score of <15.

We generated a “relapse risk score” (RRS) based on the risk of relapse with each of the following baseline variables: initial fT4, TSHR ab, gender, smoking status, and ethnicity (Figure 5). The different weightings for individual aspects of the relapse rate score were assigned according to the frequency of relapse in the indicated subgroups. The risk of relapse in those with an RRS >20 was increased by 36-fold compared to those with an RRS of <15 (OR 36.0, 95% CI 3.2–405.9, *P* = 0.004) (Figure 5).

## Discussion

Graves' disease is the commonest cause of hyperthyroidism (24), and prompt achievement of euthyroidism is a fundamental therapeutic goal (14). In the present study, we report a pharmacodynamic relationship between the dose of carbimazole and the fall in thyroid hormone levels, to aid clinicians in the rapid achievement of euthyroidism whilst avoiding the sequelae of overtreatment and hypothyroidism.

In our cohort, we observed a female preponderance (female: male ratio of ~4:1), consistent with studies undertaken in the UK (25), and other iodine replete areas (1) including Sweden (26), Denmark and Iceland (27). The mean age of 44 years of our cohort is consistent with the reported peak incidence of Graves' disease in the third to fifth decades (1, 27, 28). Patients with higher TSHR antibody titres had higher initial fT4 and fT3 levels. TSHR antibody titre is considered a marker of severity of Graves' disease, with higher titres at diagnosis being associated with a longer time to achieve euthyroid status (29).

Initial fT4:fT3 ratio was ~2:1 ratio; a measure which has been reported to provide diagnostic value to distinguish hyperthyroidism due to Graves' disease from other etiologies (30–32). Izumi and colleagues reported a similar fT4:fT3 ratio in patients with Graves' thyrotoxicosis (2.5:1), whereas patients with painless thyroiditis had a ratio of 3.3:1 (31). Serum fT3 and fT4 levels were increased and fell proportionately in response to treatment in a ~1:1 ratio. This is in contrast to the higher ratio of fT4:fT3 observed in the euthyroid state and reflects upregulation of the type 2 deiodinase enzyme with thyrotoxicosis. This data supports the use of fT4 alone in the monitoring of response to medical therapy.

This observation also explicates the phenomenon of “T3 toxicosis”—a condition occurring in <5% of all patients with hyperthyroidism, where only fT3, but not fT4, is raised above the reference range (33). As hyperthyroidism is associated with thyroidal up-regulation of type 2 deiodinase, there is an increased conversion of T4 to T3 resulting in a reduced fT4:fT3 ratio than in the euthyroid state (34). As the euthyroid reference range for fT3 is lower than that for fT4, this results in fT3 being more frequently outside the euthyroid reference range than fT4. Thus, this suggests that “T3 toxicosis” is a function of the euthyroid reference ranges and represents a mild form of Graves' disease, rather than being a distinct disease entity.

Carbimazole has a plasma half-life of ~5.3 h (35), and traditionally was prescribed in divided doses (36). However, several studies have demonstrated that a single dose of carbimazole is just as effective at inducing euthyroidism (16, 37–40). Furthermore, methimazole is concentrated in thyroid follicular tissue (41, 42) and has a longer biological duration of action than suggested by pharmacokinetic levels. Moreover, compliance is increased by once daily dosing of ATD (43). In the present study, we did not find any evidence that splitting the dose or adjusting the dose for body weight significantly altered the fall in the thyroid hormones following carbimazole.

We observed that the absolute fall in fT4 per day was directly proportional to the initial fT4 level, and thus we used a percentage fall in fT4 to investigate a pharmacodynamic relationship. Notably, a lesser reduction in percentage fall in fT4 was observed with increased duration from initial diagnosis. We identified that an interval of at least 21 days was required to reliably assess repeat fT4 levels. We had insufficient numbers of patients receiving PTU to conduct a precise dose-response relationship (*n* = 10–11 per timepoint), however patients on 200–400 mg of PTU had 1% reduction in fT4 per day, as observed with 30 mg of carbimazole, consistent with the 10-fold dosing equivalence to carbimazole reported in the literature (14, 44). Our dose-response models have acceptable fit to the data, with typical model error within 4.28 pmol/L for fT4 being 12.8% of the mean initial level of 33.4 pmol/L, and with typical model error within 2.67 pmol/L for fT3 being 13.8% of the mean initial level of 19.3 pmol/L. The models therefore provide useful indicative comparisons of the effects of different doses for patients with known baseline fT4 and fT3 levels. In this sense the models form an intermediate stage in the development of fully clinically valid dose-response models. The further work needed to derive clinically valid models is (a) the incorporation of more longitudinal data, (b) careful mixed effects analysis to determine posterior predictive *p*-values, and (c) external validation by assessing predictions for observations not used to derive the models.

Reported relapse rates following treatment with antithyroid treatment are higher compared with radioactive iodine (RAI) or surgery (45). It therefore could be advantageous to identify patients with a greater chance of relapse based on baseline characteristics to enable the stratification of patients who could best be served by definitive management with radioiodine or surgery at an earlier juncture. In our cohort, 35% of patients who completed 18 months of treatment with antithyroid therapy experienced subsequent relapse. The risk was increased in men, white British ethnicity, smokers, and those with higher fT4 and TSHR ab titres. Some reports have suggested that smoking could modulate the immune response to make relapse more likely (46, 47), although the exact mechanism remains elusive. Similarly, patients with a final serum fT4 in the upper range of normal (≥15 pmol/l) were more likely to experience relapse. An adjusted multivariable logistic regression found that only initial fT4 predicted the odds of relapse.

In a prospective, observational, multi-centre study, similar rates of relapse to our study were reported, with 37% of 178 patients experiencing recurrent Graves' hyperthyroidism within 2 years after withdrawal of ATD. Younger age <40 yrs, higher serum fT4 level >40 pmol/l, higher serum TBII titre, larger goiter size at diagnosis, and genetic factors including PTPN22 C/T polymorphisms, and HLA subtypes DQB1^*^02, DQA1^*^05, and DRB1^*^03 were independent risk predictors for recurrence (48). Based on these clinical markers, the GREAT (Graves Recurrence Events After Therapy) score was able to differentiate the risk of relapse into three groups, whereby 68% of patients in the highest risk group relapsed (vs. 16% in the lowest risk group) and all did so rapidly within 6 months (48). Our proposed relapse rate score appears to perform similarly well and incorporates comparable variables, however external validation, as discussed above, is required to validate this proposed score.

There is a paucity of data describing the relationship between carbimazole dose and the subsequent change in thyroid hormone levels. Thus, these data represent an important first step in establishing the relationship between dose and pharmacodynamic response to carbimazole. However, it is necessary to stress that as the models were developed using retrospective data, external validation of the predictions outlined in the present study is required prior to consideration for clinical use.

In summary, we have identified a pharmacodynamic relationship for medical therapy of Graves' disease with dose of carbimazole. The titration approach is desirable as it facilitates reduced cumulative exposure to ATDs and thus carries a lower risk of dose-related side effects. Whilst Graves' disease is one of the most common endocrine diseases treated, the pharmacodynamic response to carbimazole has been poorly defined, causing a residual significant risk of under- or over-treatment. Thus, the relationships described in the present study could aid in dose-selection and represents a useful initial step to the estimation of factors that influence the response to ATD therapy.

## Data Availability Statement

The datasets generated for this study are available on request to the corresponding author.

## Ethics Statement

Ethical review and approval was not required for the study on human participants in accordance with the local legislation and institutional requirements. Written informed consent for participation was not required for this study in accordance with the national legislation and the institutional requirements.

## Author Contributions

AA, SC, ASa, FW, AC, KM, TK, and WD designed the study, analyzed the data, prepared the manuscript, and designed the figures and tables. AA, SC, RB, ASi, PE, MP, DP, and CI-E conducted data collection. AA, SC, RB, AS, EJ, and TK performed the statistical analysis. WD was the project supervisor, reviewed and edited the manuscript, and is the guarantor of this research project. All authors have made a substantial, direct and intellectual contribution to the work and approved the manuscript prior to its submission.

## Conflict of Interest

The authors declare that the research was conducted in the absence of any commercial or financial relationships that could be construed as a potential conflict of interest.

## Supplementary Material

Supplemental Figure 1 **(A)** Bar graph of absolute fall in fT4 per day in patients on carbimazole following their initial visit with groups compared using Kruskal-Wallis test with *post-hoc* Dunn's test (^*^*P* < 0.05, ^**^*P* < 0.01, ^***^*P* < 0.001, ^****^*P* < 0.0001). **(B)** Bar graph of absolute fall in fT4 per day in patients on carbimazole following their follow-up visit with groups compared using Kruskal-Wallis test with *post-hoc* Dunn's test (^*^*P* < 0.05, ^**^*P* < 0.01, ^***^*P* < 0.001, ^****^*P* < 0.0001). **(C)** Scattergraph of median absolute fall in fT4 per day in patients receiving carbimazole at both initial and follow-up visits. Initial median absolute fall in fT4 per day = 0.01^*^ carbimazole dose −0.01, *r*^2^ = 0.97, *P* = < 0.0001. Follow-up median absolute fall in fT4 per day = 0.007^*^ carbimazole dose −0.05, *r*^2^ = 0.99, *P* ≤ 0.0001. **(D)** Bar graph of absolute fall in fT3 per day in patients on carbimazole following their initial visit with groups compared using Kruskal-Wallis test with *post-hoc* Dunn's test (^*^*P* < 0.05, ^**^*P* < 0.01, ^***^*P* < 0.001, ^****^*P* < 0.000). **(E)** Bar graph of absolute fall in fT3 per day in patients on carbimazole following their follow-up visit with groups compared using Kruskal-Wallis test with *post-hoc* Dunn's test (^*^*P* < 0.05, ^**^*P* < 0.01, ^***^*P* < 0.001, ^****^*P* < 0.0001). **(F)** Scattergraph of median absolute fall in fT3 per day in patients receiving carbimazole at both initial and follow-up visits. Initial median absolute fall in fT3 per day = 0.01^*^carbimazole dose −0.02, *r*^2^ = 0.87, *P* = 0.0015. Follow-up median absolute fall in fT3 per day = 0.003^*^carbimazole dose – 0.03, *r*^2^ = 0.94, *P* = 0.0015.

Supplemental Table 1 Dose-response coefficients. Weight dependent values of percentage slope and intercept for use in the models for fT4 and fT3 fall given a known dose. Entries are divided by 100 before use in the respective formula to obtain absolute rather than percentage falls.
