# Supplementary figures and images for: Pharmacodynamic Response to Anti-thyroid Drugs in Graves' Hyperthyroidism
Source: Front Endocrinol (Lausanne). 2020 May 12;11:286. doi: 10.3389/fendo.2020.00286 (PMC7236601; doi:10.3389/fendo.2020.00286)

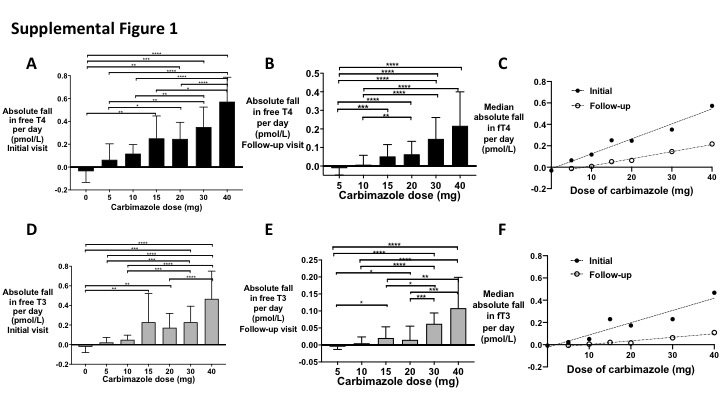

Supplement: Supplemental Figure 1 — (A) Bar graph of absolute fall in fT4 per day in patients on carbimazole following their initial visit with groups compared using Kruskal-Wallis test with post-hoc Dunn's test (*P < 0.05, **P < 0.01, ***P < 0.001, ****P < 0.0001). (B) Bar graph of absolute fall in fT4 per day in patients on carbimazole following their follow-up visit with groups compared using Kruskal-Wallis test with post-hoc Dunn's test (*P < 0.05, **P < 0.01, ***P < 0.001, ****P < 0.0001). (C) Scattergraph of median absolute fall in fT4 per day in patients receiving carbimazole at both initial and follow-up visits. Initial median absolute fall in fT4 per day = 0.01* carbimazole dose −0.01, r2 = 0.97, P = < 0.0001. Follow-up median absolute fall in fT4 per day = 0.007* carbimazole dose −0.05, r2 = 0.99, P ≤ 0.0001. (D) Bar graph of absolute fall in fT3 per day in patients on carbimazole following their initial visit with groups compared using Kruskal-Wallis test with post-hoc Dunn's test (*P < 0.05, **P < 0.01, ***P < 0.001, ****P < 0.000). (E) Bar graph of absolute fall in fT3 per day in patients on carbimazole following their follow-up visit with groups compared using Kruskal-Wallis test with post-hoc Dunn's test (*P < 0.05, **P < 0.01, ***P < 0.001, ****P < 0.0001). (F) Scattergraph of median absolute fall in fT3 per day in patients receiving carbimazole at both initial and follow-up visits. Initial median absolute fall in fT3 per day = 0.01*carbimazole dose −0.02, r2 = 0.87, P = 0.0015. Follow-up median absolute fall in fT3 per day = 0.003*carbimazole dose – 0.03, r2 = 0.94, P = 0.0015. [file Image_1.jpg]
