# Supplementary material for: Pharmacodynamic Response to Anti-thyroid Drugs in Graves' Hyperthyroidism
Source: Front Endocrinol (Lausanne). 2020 May 12;11:286. doi: 10.3389/fendo.2020.00286 (PMC7236601; doi:10.3389/fendo.2020.00286)
Supplement: Supplemental Table 1 — Dose-response coefficients. Weight dependent values of percentage slope and intercept for use in the models for fT4 and fT3 fall given a known dose. Entries are divided by 100 before use in the respective formula to obtain absolute rather than percentage falls. [file Table_1.docx]

**Supplemental Table 1 – Dose-response coefficients.**

| Body Weight on presentation (kg) | fT4 slope | fT4 intercept | fT3 slope | fT3 intercept |
| --- | --- | --- | --- | --- |
| <50 | 0.08 | 0.17 | 0.12 | -0.27 |
| 50 - 60 | 0.06 | 0.4 | 0.11 | -1.0 |
| 60 - 70 | 0.07 | 0.04 | 0.08 | 0.01 |
| 70 - 100 | 0.06 | -0.04 | 0.1 | -0.15 |
| >100 | 0.06 | -0.04 | 0.06 | -0.17 |

**Supplemental Table 1 – Dose-response coefficients.** Weight dependent values of percentage slope and intercept for use in the models for fT4 and fT3 fall given a known dose. Entries are divided by 100 before use in the respective formula to obtain absolute rather than percentage falls.
